# Integrated genetic and methylomic analyses identify shared biology between autism and autistic traits

**DOI:** 10.1186/s13229-019-0279-z

**Published:** 2019-07-17

**Authors:** Aicha Massrali, Helena Brunel, Eilis Hannon, Chloe Wong, iPSYCH-MINERvA Epigenetics Group, Simon Baron-Cohen, Varun Warrier

**Affiliations:** 1 Autism Research Centre, University of Cambridge, Cambridge, UK; 2 University of Exeter Medical School, University of Exeter, RILD Building, Level 4, Barrack Rd, Exeter, UK; 3 King’s College London, Institute of Psychiatry, Psychology and Neuroscience, De Crespigny Park, London, UK; 4 Cambridgeshire and Peterborough National Health Service Foundation Trust, Cambridge, UK

## Abstract

Previous studies have identified differences in DNA methylation in autistic individuals compared to neurotypical individuals. Yet, it is unclear if this extends to autistic traits—subclinical manifestation of autism features in the general population. Here, we investigate the association between DNA methylation at birth (cord blood), and scores on the Social and Communication Disorders Checklist (SCDC), a measure of autistic traits, in 701 8-year-olds, by conducting a methylome-wide association study (MWAS). We did not identify significant CpGs associated with SCDC. The most significant CpG site was cg14379490, on chromosome 9 (MWAS beta = − 1.78 ± 0.35, *p* value = 5.34 × 10^*−7*^). Using methylation data for autism in peripheral tissues, we did not identify a significant concordance in effect direction of CpGs with *p* value < 10^−4^ in the SCDC MWAS (binomial sign test, *p* value > 0.5). In contrast, using methylation data for autism from post-mortem brain tissues, we identify a significant concordance in effect direction of CpGs with a *p* value < 10^−4^ in the SCDC MWAS (binomial sign test, *p* value = 0.004). Supporting this, we observe an enrichment for genes that are dysregulated in the post-mortem autism brain (one-sided Wilcoxon rank-sum test, *p* value = 6.22 × 10^−5^). Finally, integrating genome-wide association study (GWAS) data for autism (*n* = 46,350) with mQTL maps from cord-blood (*n* = 771), we demonstrate that mQTLs of CpGs associated with SCDC scores at *p* value thresholds of 0.01 and 0.005 are significantly shifted toward lower *p* values in the GWAS for autism (*p* < 5 × 10^−3^). We provide additional support for this using a GWAS of SCDC, and demonstrate a lack of enrichment in a GWAS of Alzheimer’s disease. Our results highlight the shared cross-tissue methylation architecture of autism and autistic traits, and demonstrate that mQTLs associated with differences in DNA methylation associated with childhood autistic traits are enriched for common genetic variants associated with autism and autistic traits.

**Electronic supplementary material:**

The online version of this article (10.1186/s13229-019-0279-z) contains supplementary material, which is available to authorized users.

## Introduction

Autism is a neurodevelopmental condition characterized by social-communication difficulties, unusually restrictive, repetitive behavior and narrow interests, and sensory difficulties [1, 2]. The condition can be thought as a continuum, with autistic traits being normally distributed in the general population, and autism at the extreme end of the continuum [3–5]. Both autism and autistic traits are highly heritable [6–9], with variation across the allelic spectrum associated with the condition [10–12]. Despite a significant SNP heritability (Autism: h^2^_SNP_ – 0.49 [12] – 0.12 [11]), recent studies have demonstrated that the variance explained per SNP is small, suggesting a highly polygenic architecture [11, 13]. None of the significant SNPs associated with autism alter protein coding, suggesting that gene expression is regulated through other mechanisms [11, 14]. For instance, a recent genome-wide association study (GWAS) of autism has identified an enrichment of GWAS signals in H3K4me1 histone marks, particularly in brain and neural cell lines [11, 13].

Previous studies have investigated autism associated methylation signatures in both peripheral tissues [14–16] (50 < *N* < 2917) and in the post-mortem brain [17–20] (31 < *N* < 81). While post-mortem brain is pertinent for a neurodevelopmental condition like autism, it is not readily accessible, and will be confounded by post-mortem effects on DNA methylation. Studies of methylation signatures in post-mortem brains in autism have replicably identified differential methylation [17–20]. Further, they have demonstrated an enrichment for differentially methylated signatures in the immune system, synaptic signaling, and neuronal regulation [17, 18, 20]. In contrast, recent large-scale analysis of three different peripheral tissue datasets have not identified significantly differentially methylated CpG sites in autistic individuals compared to typically developing individuals [14, 16]. The lack of significant results in peripheral tissues may be attributable to small effect sizes, and significant heterogeneity in both CpG methylation and autism.

While a few studies have investigated DNA methylation underlying autism, to our knowledge, there has been no study investigating DNA methylation underlying autistic traits in the general population, which are subthreshold manifestations of the autism phenotype. One measure of autistic traits is the Social and Communication Disorders Checklist (SCDC) [21]. Scores on the SCDC are associated (Cohen’s *D* = 2.8, *p* value < 0.001) [21] and genetically correlated with autism (*r*_g_ ~ 0.3) [5, 21, 22]. The SCDC has a modest SNP heritability (h^2^_SNP_ = 0.24, s.e. = 0.07) [23], and polygenic scores for autism are associated with SCDC scores in the general population (max *R*^2^ = 0.13%) [22]. An advantage of using a continuous measure of autistic traits is that it captures the underlying variance better, and minimizes heterogeneity attributable to different diagnostic criteria and practices used to diagnose autism.

One potential mechanism through which common genetic variants can regulate gene expression is through DNA methylation. DNA methylation is partly heritable (0.05 < h^2^_twin_ < 0.19, defined as the proportion of variance in methylation that is attributable to genetics) [24–26]. A few studies have integrated genetics and methylation to identify convergent signatures in autism. Andrews and colleagues demonstrated that autism associated GWAS loci are enriched for methylation QTLs (mQTLs) in fetal brain and blood, suggesting that at least some of the genetic loci associated with autism may contribute to the condition through differential methylation [27]. In line with this, Hannon and colleagues demonstrated that polygenic risk for autism is associated with differential methylation at birth [14]. While these studies have demonstrated a role for common genetic variants associated with autism and influencing methylation, to our knowledge no study has investigated if methylation of CpGs associated with autistic traits are enriched for common genetic variants associated with autism or autistic traits. One way to test this hypothesis is using mQTLs. We hypothesized that mQTLs of significant CpGs in a methylome-wide association study (MWAS) of autistic traits will be enriched for lower *p* values in a GWAS of autism or autistic traits.

To address these questions, we investigated the association of CpG methylation in cord blood using scores on the SCDC at age 8. The use of cord blood CpGs minimizes (though, does not eliminate) reverse causation (where the phenotype influences DNA methylation), as the methylation of CpG sites is measured very early in life. To investigate how comparable an MWAS of an autistic trait is to other MWAS of autism and related phenotypes conducted across different tissues, we investigated the overlap between the MWAS of SCDC and other MWAS of autism and communication-related traits in peripheral and post-mortem brain tissues. We further investigated if genes that are transcriptionally dysregulated in the post-mortem autism brain are enriched for methylation CpGs associated with SCDC. Finally, integrating GWAS data for autism from 46,350 individuals, we investigated if mQTLs of CpGs associated with SCDC scores at various *p* value thresholds are significantly shifted toward lower *p* values in the autism GWAS. We validated these results using a smaller GWAS for SCDC.

In summary, this study had two specific aims: (1) to investigate if an MWAS for autistic traits identifies significant CpG methylation and if it is comparable to MWAS of autism; and (2) to investigate if mQTLs of CpGs associated with autistic traits at various *p* value thresholds are enriched in GWAS of autism and autistic traits.

## Methods

### Participants

Participants were children from the Accessible Resource for Integrated Epigenetic Studies (ARIES, www.ariesepigenomics.org.uk) [28], a subset of the Avon Longitudinal Study of Children and Parents (ALSPAC) [29]. Methylation data was only available for participants in the ARIES substudy. ALSPAC is a longitudinal cohort in which the participants were pregnant women in the Avon region in the UK. The initial cohort consists of 14,541 initial pregnancies and 13,988 children who were alive at the age of 1. In addition, children were enrolled in further phases. Details of the data available can be found on the online data dictionary here: http://www.bristol.ac.uk/alspac/researchers/access/. Written informed consent was obtained from the parent or the guardian of the child and assent was obtained from the child where possible. The study was approved by the ALSPAC Ethics and Law committee, and the Cambridge Human Biology Research Ethics Committee.

The participants of the primary MWAS of SCDC were 701 children who completed the SCDC at age 8, and for whom epigenetic data was available (341 males and 360 females). Of the participants included in the primary MWAS (SCDC), only five participants had an autism diagnosis based on a parental questionnaire at 9.5 years of age. We conducted a secondary MWAS of pragmatic communication in 666 children. Pragmatic communication was measured using the Children’s Communication Checklist [30] (CCC) at age 9 (323 males and 340 females). In addition, we conducted a GWAS of SCDC scores in a sample of 5,628 8-year-olds from ALSPAC, details of which are provided below. This sample included participants who were included in the two MWAS (SCDC and CCC).

### Phenotypic measures

The SCDC is a 12-item questionnaire that measures difficulties in verbal and nonverbal communication and social interaction including reciprocal interaction [21]. Scores range from 0 to 24 with high scores reflecting difficulties in social interaction and communication. The SCDC has good psychometric properties—internal consistency of 0.93 and test-retest reliability of 0.81 [21]. We used mother-reported SCDC scores on children aged 8. The mean of SCDC scores in our sample was 14.65 (standard deviation = 3.44). Previous research has demonstrated that the SCDC is stable over time and scores at different ages are genetically correlated [22, 31]. SNP heritability is highest for  SCDC scores in  childhood (at the age of 91 months) and in later adolescence (17 years) [22, 31]. We focused on SCDC scores at 91 months as the sample size was the largest, has highest genetic correlation with autism [22], and the exposure to environmental factors is limited at 91 months compared to other time points.

A second measure that we used in this study is the 53-item parent-completed CCC which measures pragmatic communication [30]. The CCC and subscales have moderate to high twin heritability [32], and moderate SNP heritability (h^2^_SNP_ = 0.18) [23]. There is a negative correlation between the CCC and the SCDC [33]. The mean of the CCC in the sample of 666 children was 151.83 (standard deviation = 6.77), with scores ranging from 111 to 162. To make the analysis comparable with the SCDC (which measures difficulties rather than ability), we reverse scored the CCC so that higher scores measure difficulties in pragmatic communication.

The histograms of both the phenotypes in the samples used in the study are provided in Additional file 1: Figure S1. We calculated the phenotypic correlation between the CCC and the SCDC in the samples used in this study using Pearson’s correlation.

### Cord blood DNA methylation, quality control, and normalization

Array-based cord blood methylation quantification was conducted by ARIES [28]. Briefly, DNA was extracted from cord blood drawn from the umbilical cord upon delivery. Following extraction, DNA was bisulfite-converted using the Zymo EZ-DNA MethylationTM kit (Zymo, Irvine, CA). Then, methylation of over 485,000 CpG sites was measured using the Illumina HumanMethylation450 BeadChip array according to the standard protocol. The arrays were scanned using an Illumina iScan and initial quality review was assessed using GenomeStudio (version 2011.1).

Methylation assays utilize a pair of probes to detect methylation of cytosine at CpG sites. One is used to detect methylated loci (M) and the other is used to detect unmethylated CpG islands (U). The level of methylation at a locus is then estimated based on the ratio of signals from M to U, called “beta” value. Beta values range from 0 (no cytosine methylation) to 1 (complete cytosine methylation). Sample information and participant demographics are provided in Additional file 1: Table S1.

### QC and normalization

In total, there were 1,127 cord blood samples including technical replicates (i.e., samples that were of poor quality with low detection score and were thus repeated). Of these, 241 were from blood spots and 886 were from white cells. Blood spots were obtained from cord blood and not taken from heel prick. The provided data was quality controlled by ARIES team. The QC procedure employed by the ARIES team includes removing participants who did not pass mother-child genotype-based relatedness control, participants who were outliers for genetic heterozygosity, genetic ethnicity outliers, samples with low bead numbers, and detection *p* value > 0.05 (probability that the target sequence signal was distinguishable from the background). This resulted in a total of 914 participants. None of these participants had a sex mismatch, where the genetic sex was different from reported sex. We further removed nine duplicate samples, resulting in 905 participants. Further, 782 of these participants had phenotypic data on the SCDC. Finally, we removed 81 related individuals, resulting in a final sample of 701 participants who had both methylation and phenotypic data.

The data was normalized using functional normalization implemented in the R package meffil (https://github.com/perishky/meffil) [34]. Functional normalization is a between-array normalization method for the Illumina Infinium HumanMethylation450 platform and an extension of quantile normalization. It removes unwanted technical variation. The normalization procedure was performed to the methylated and unmethylated signal intensities, and type I and type II probes separately. For X and Y chromosomes, males and females were normalized separately using the sex at birth information.

We removed CpG sites whose probe or single-base extension overlaps with a SNP with MAF > 0.01. We further removed cross-reactive probes identified in Chen et al. 2013 [35] as implemented in meffil. In total, 372,662 CpG sites remained after quality control. Cell proportions for CD4 T lymphocytes, CD8 T lymphocytes, B lymphocytes, natural killer cells, monocytes, and granulocytes were estimated using the minfi package [36]. These cell types were estimated using post hoc procedures as cell type information was not collected prior to DNA extraction, further details of which are provided elsewhere [28].

### Methylome-wide association

A methylome-wide association study was run using a two-step regression model (model 1). In the first regression, normalized epigenetic probe betas were regressed against technical covariates (slide, sample type, i.e., white blood cells vs blood spots, and plate and cell counts), using the following model.

1 $$ {\beta eta}_{meth}={\beta}_0+{\beta}_1\mathrm{Slide}+\kern0.5em {\beta}_2\mathrm{Sample}+{\beta}_3\mathrm{Plate}+{\beta}_{4\dots n}\mathrm{Cellcount}+\varepsilon $$

The residuals from this regression were further used as corrected methylation values. In the second regression, SCDC (or CCC) scores were regressed against corrected methylation values with sex and the first two genetic principal components as covariates, as provided below:

2 $$ \mathrm{SCDC}={\beta}_0+{\beta}_1\varepsilon +\kern0.5em {\beta}_2 Sex+{\beta}_3{PC}_1+{\beta}_4{PC}_2+\zeta $$

We did not correct for age as methylation was measured at birth, and SCDC was measured at 8 years of age for all participants. Here, we were specifically testing if methylation status measured in cord blood was associated with autistic traits or pragmatic language measured at a later age. Given the highly skewed distribution of the SCDC scores, we used a negative binomial regression, using the MASS package in R, which involves by default applying a chi-square test to validate the model (goodness of fit test). We used a Bonferroni-corrected epigenome-wide significant threshold of 1 × 10^−7^ to identify significant associations. All analyses were conducted in R version 3.2. A Pearson correlation test between both regression coefficients and Z-score (regression Beta divided by the standard error) from both SCDC and CCC MWAS models was performed to assess the epigenetic correlation between both traits.

To evaluate that the results are robust to methodological differences, we used a second model to conduct the methylome-wide association (model 2). This too was conducted using a two-step regression. Here, in the first regression, we used the *M* value [37] rather than the methylation beta value, and regressed it against cell counts generated using “GSE68456” [38] which includes nucleated red blood cells in cord blood. Thus, our first regression in model 2 is of the form:

3 $$ M-{\mathrm{value}}_{meth}={\beta}_0+{\beta}_1\mathrm{Slide}+\kern0.5em {\beta}_2\mathrm{Sample}+{\beta}_3\mathrm{Plate}+{\beta}_{4\dots n}\mathrm{Cellcount}+\varepsilon $$

Where the cell counts include CD4 T lymphocytes, CD8 T lymphocytes, B lymphocytes, natural killer cells, monocytes, granulocyte, and nucleated RBCs. The residuals from this regression were regressed against SCDC scores using Eq. 2 outlined above. We then evaluated if the Z-scores varied substantially between the two models using a Pearson correlation test. Throughout the manuscript, we report the results from the first model because (1) beta values are easier to interpret than M-values, and (2) only 241 participants included in the MWAS had nucleated RBC count different from 0.

To identify gene sets and networks that were differentially methylated in the SCDC MWAS, we used mGSEA [39] and used Gene Ontology-based gene sets.

**Fig. 1:**
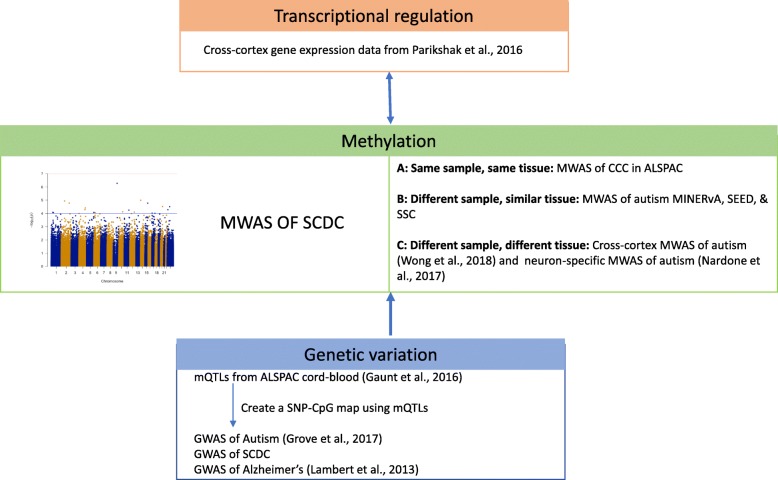
Schematic diagram of the study design. Schematic diagram of the study design

In order to interpret results from the MWAS, we designed a multi-step enrichment strategy including (1) a same-sample, same-tissue overlap and correlation analyses between the SCDC and the CCC; (2) a cross-tissue overlap analysis between the SCDC MWAS and MWAS of autism in peripheral blood and post-mortem brain tissue; (3) enrichment for autism transcriptionally dysregulated genes; and (4) enrichment of CpG-associated mQTLs in autism and SCDC GWAS. A summary of the study design is provided in Fig. 1.

### Peripheral tissue (blood and blood-spot) overlap analysis

We had access to summary MWAS statistics from three peripheral tissue datasets described in detail elsewhere (SEED [16], Simons Simplex Collection (SSC) [16], and MINERvA [14]). For all overlap analyses, we conducted two statistical tests. In the first, we tested if all nominally significant CpGs (*p* value < 0.01) in the three peripheral tissue MWAS (SEED, SSC, and MINERvA) have a shift toward lower *p* values in the SCDC MWAS (one-sided Wilcoxon rank-sum test). This tests a larger number of CpGs and is consistent with the idea that each individual CpG contributes minimally to the phenotype suggesting a polymethylomic (or, polyepigenetic) architecture similar to a polygenic architecture of complex traits. In addition, this does not test effect direction as effect direction may vary based on number of factors including tissue source. In the second analysis, we investigated effect direction concordance for CpGs with *p* value < 1 × 10^−4^ in either of the two MWAS being tested, thus, conducting 12 binomial sign tests tests in total. This restricts the analyses to a relatively small number of CpGs.

### Post-mortem brain tissue overlap analysis

We had access to summary statistics from two post-mortem brain tissue datasets to investigate overlap with the SCDC MWAS. MWAS in both these datasets were conducted using the Illumina HumanMethylation450 BeadChip making the MWAS comparable to the SCDC MWAS. We used a recent MWAS conducted using tissue from 38 idiopathic autistic individuals and 38 controls [20]. Further details are provided elsewhere [20]. To investigate if there was an overlap between the SCDC MWAS and a neuron-specific MWAS in post-mortem autism brains, we used summary MWAS data from FACS-sorted neurons in brain samples from 15 autistic individuals and 16 typical controls. Further details are provided elsewhere [17]. We did not have access to social communication data in the two post-mortem samples, making this comparison impossible.

For both datasets, our analysis was similar to the analysis of peripheral tissue MWAS. We investigated effect direction concordance between the two post-mortem brain autism MWAS and the SCDC MWAS for all CpGs with *p* value < 1 × 10^−4^ in the post-mortem brain MWAS (binomial sign test). Additionally, we investigated if CpGs with *p* value < 0.01 in either of the two post-mortem brain MWAS had a significant shift toward lower *p* values in the SCDC MWAS (one-sided Wilcoxon rank-sum test).

### Enrichment with autism-associated transcriptionally dysregulated genes

For enrichment analyses with transcriptionally dysregulated gene expression data, we used an RNA-sequencing study of 167 post-mortem cortical samples with *n* = 85 with a diagnosis of autism and *n* = 82 from nonpsychiatric controls. Samples were from BA9 (prefrontal cortex), or BA41/42 (temporal cortex) [40]. Significantly dysregulated genes had a Benjamini-Hochberg adjusted FDR < 0.05. We conducted enrichment analyses using a one-sided Wilcoxon rank-sum test. We first mapped the CpGs to genes using the CpG to gene annotation for the Illumina 450k methylation array using the *IlluminaHumanMethylation450k.db* package in R (http://www.bioconductor.org/packages/release/data/annotation/html/IlluminaHumanMethylation450kprobe.html). We restricted our analysis only to CpGs that were mapped onto the genes tested for differential expression in the post-mortem brain dataset [40]. We then compared the distribution of the SCDC *p* values for CpGs mapped to significantly differentially dysregulated genes vs the other genes.

### Enrichment of CpG-associated mQTLs in GWAS of autism and SCDC

We investigated if mQTLs of CpGs below four *p* value thresholds in the SCDC MWAS (P_SCDC_) had lower *p* values compared to other mQTLs in the GWAS (P_GWAS_) of (1) autism, (2) SCDC, and (3) as a negative control, Alzheimer’s. We hypothesized that the mQTLs of CpGs below P_SCDC_ will have significantly lower P_GWAS_ in comparison with remaining mQTLS. To map CpGs to mQTLS, we used mQTL maps generated by the ARIES cohort in cord blood (*n* = 771), restricting our analysis to only significant mQTLS identified after FDR correction (*p* value < 0.05 after FDR correction) [41]. This cohort overlapped with the sample in which the MWAS was conducted. All mQTLs had a minor allele frequency > 1%. For each CpG-mQTL pair, we restricted our analysis to only those CpG-mQTL pairs investigated in both the SCDC MWAS and the GWAS of interest. In other words, the CpGs must have been investigated in the SCDC MWAS and the paired mQTL of the CpG must have been investigated in the GWAS of interest. As none of the CpGs meet the strict *p* value threshold, we had to use several thresholds at different levels of stringency. To control the signal-to-noise ratio in the context of an MWAS, we considered four empirical P_SCDC_ thresholds: 0.05, 0.01, 0.005, and 0.001. Enrichment was conducted using permutation testing, where we defined 10,000 null sets. We identified three potential factors that may influence this analysis: (1) the linkage disequilibrium (LD) structure of mQTLs, (2) the number of mQTLs mapped onto a CpG, and (3) the number of CpGs a single mQTL is mapped onto. To address LD, first, we clumped the list of mQTLs using an *r*^2^ of 0.6 and distance of 1000 kb, to ensure that linkage disequilibrium among these mQTLs does not confound the outcome. In this clumped list of mQTLs, the majority were mapped to only one mQTL. Second, to account for the number of mQTLs mapped onto CpGs, we binned the CpGs into six groups based on the number of SNPs they mapped onto (1–5, 6–10, 11–15, 15–20, 20–25, and above 25), and conducted enrichment analysis so that every mQTL in the null set matched the original mQTL based on CpG bins. Third, one single mQTL may map onto multiple CpGs, resulting in non-unique CpG-mQTL pairs with P_SCDC_ < threshold, and P_SCDC_ > threshold. We retained unique CpG-mQTL pairs in each list before conducting permutation-based enrichment analysis. Finally, to account for multiple testing, as we tested across four non-independent *p* value thresholds, the empirical *p* values were corrected for the four tests using Benjamini-Hochberg FDR correction. Empirical *p* values were significant at FDR < 0.05.

We examined the results identified in the Autism GWAS using a GWAS of log-transformed SCDC scores in ALSPAC (details below). As a negative control, we used GWAS data for Alzheimer’s (phase I), downloaded from IGAP (http://web.pasteur-lille.fr/en/recherche/u744/igap/igap_download.php) [42], and tested for enrichment using an identical procedure as mentioned above. The Alzheimer’s GWAS (phase I, for which genome-wide summary data is available) consists of 17,008 cases and 37,154 controls, and identified 14 significant GWAS loci. While both autism and Alzheimer’s are neuropsychiatric conditions, the genetic correlation between the two conditions is non-significant (*r*_g_ = 0.04 ± 0.10; *p* value = 0.102), suggesting minimal shared genetics. The number of cases and controls used in the two studies (phase 1 for the Alzheimer’s GWAS) are comparable, providing approximately similar statistical power (mean chi-square: Alzheimer’s = 1.114, autism = 1.2). Further, they are distinct in that autism is a neurodevelopmental condition diagnosable at childhood, while Alzheimer’s is largely diagnosed in individuals who are 65 or older.

### GWAS of SCDC scores

We conducted a log-transformed GWAS of SCDC scores at age 8 in the ALSPAC data. Note that log-transformed phenotype models are computationally more efficient for high-dimensional GWAS data than negative binomial models used in the MWAS. Further, we identified a high correlation between the log-transformed SCDC MWAS and the negative binomial SCDC MWAS (*r*_Beta_ = 0.98, P 2.2 × 10^−16^; *r*_Zscores_ = 0.99, P 2.2 × 10^−16^), suggesting that the results are almost identical between the two statistical models. Participants were genotyped using the Illumina® HumanHap550 quad chip by Sample Logistics and Genotyping Facilities at Wellcome Sanger Institute and LabCorp (Laboratory Corporation of America) using support from 23andMe. We restricted our analysis only to individuals of European descent. This was identified using multidimensional scaling analysis and compared with Hapmap II (release 22) [43]. We excluded individuals with sex mismatches, high missingness (> 3%), and disproportionate heterozygosity, and if cryptic relatedness, identified using identity by descent, was greater than 0.1. We removed SNPs with greater than 5% missingness, those that violated Hardy-Weinberg equilibrium (*p* value < 1 × 10^−6^), and those with a minor-allele frequency less than 1%. This resulted in a total of 526,688 genotyped SNPs. Haplotypes were estimated using data from mothers and children using ShapeIT (v2.r644) [44]. Imputation was performed using Impute2 V2.2.2 against the 1000 genomes reference panel (Phase 1, Version 3) [45]. Imputed SNPs were excluded from all further analyses if they had a minor allele frequency < 1% and info < 0.8. After quality control, there were 8,282,911 genotyped and imputed SNPs that were included in subsequent analyses. GWAS analysis was conducted for mother-reported SCDC scores at age 8 that was log-transformed given the highly skewed distribution. Linear regression was conducted in Plink v1.9 [46] that converted allele dosages into hard calls. We included the first two ancestry principal components and sex as covariates in the regression model. The first two ancestry principal components were calculated using Plink 1.9 in unrelated individuals, using SNPs with MAF > 5% that were pruned for LD (*r*^2^ < 0.1).

As reported previously [5, 22, 31], the SNP heritability as quantified using LDSC [47, 48] was *h*^2^ = 0.12 ± 0.05. The LDSR intercept (0.99) suggested that there was no inflation in GWAS estimates due to population stratification. The λ_GC_ was 1.013. We replicated the previously identified genetic correlation (constrained intercept) [5] with autism using our SCDC GWAS (PGC-autism: *r*_g_ = 0.46 ± 0.20, *p* value = 0.019; iPSYCH-autism: *r*_g_ = 0.45 ± 0.18, *p* value = 0.01).

### Data, software, and script availability:

- MWAS summary statistics:
  The summary statistics for the MWAS (SCDC and CCC) can be downloaded from here: https://www.dropbox.com/sh/8za5xspmbjydpst/AAA_ZGmMLOE8Ql7egi5Mcu8Ha?dl=0 . These summary statistics are also provided as RData files with this manuscript.
  Summary statistics for the SEED and the SSC MWAS can be obtained from here: https://molecularautism.biomedcentral.com/articles/10.1186/s13229-018-0224-6 .
  Summary statistics for the MINERvA cohort can be obtained by contacting Jonas Bybjerg-Grauholm.
- GWAS summary statistics:
  The summary statistics for the autism GWAS (iPSYCH) can be downloaded from http://www.med.unc.edu/pgc/results-and-downloads (iPSYCH-PGC GWAS-2017).
  The Alzheimer’s GWAS can be downloaded from http://web.pasteur-lille.fr/en/recherche/u744/igap/igap_download.php.
  The summary statistics for the SCDC GWAS can be obtained from https://www.dropbox.com/sh/8za5xspmbjydpst/AAA_ZGmMLOE8Ql7egi5Mcu8Ha?dl=0.
- Scripts for running the two regression models for the MWAS and running the enrichment analyses with the mQTL data are available here: https://github.com/autism-research-centre/MWAS_autistictraits
- mQTL data used in this (coord blood) is a part of the ARIES cohort, and can be downloaded here: http://www.mqtldb.org/
- We used the following software/packages: Plink (http://zzz.bwh.harvard.edu/plink/); IlluminaHumanMethylation450k.db (http://www.bioconductor.org/packages/release/data/annotation/html/IlluminaHumanMethylation450kprobe.html); MASS (https://cran.r-project.org/web/packages/MASS/index.html); LDSC (https://github.com/bulik/ldsc/wiki/Heritability-and-Genetic-Correlation).

## Results

### Methylome-wide association study of the SCDC scores

**Fig. 2:**
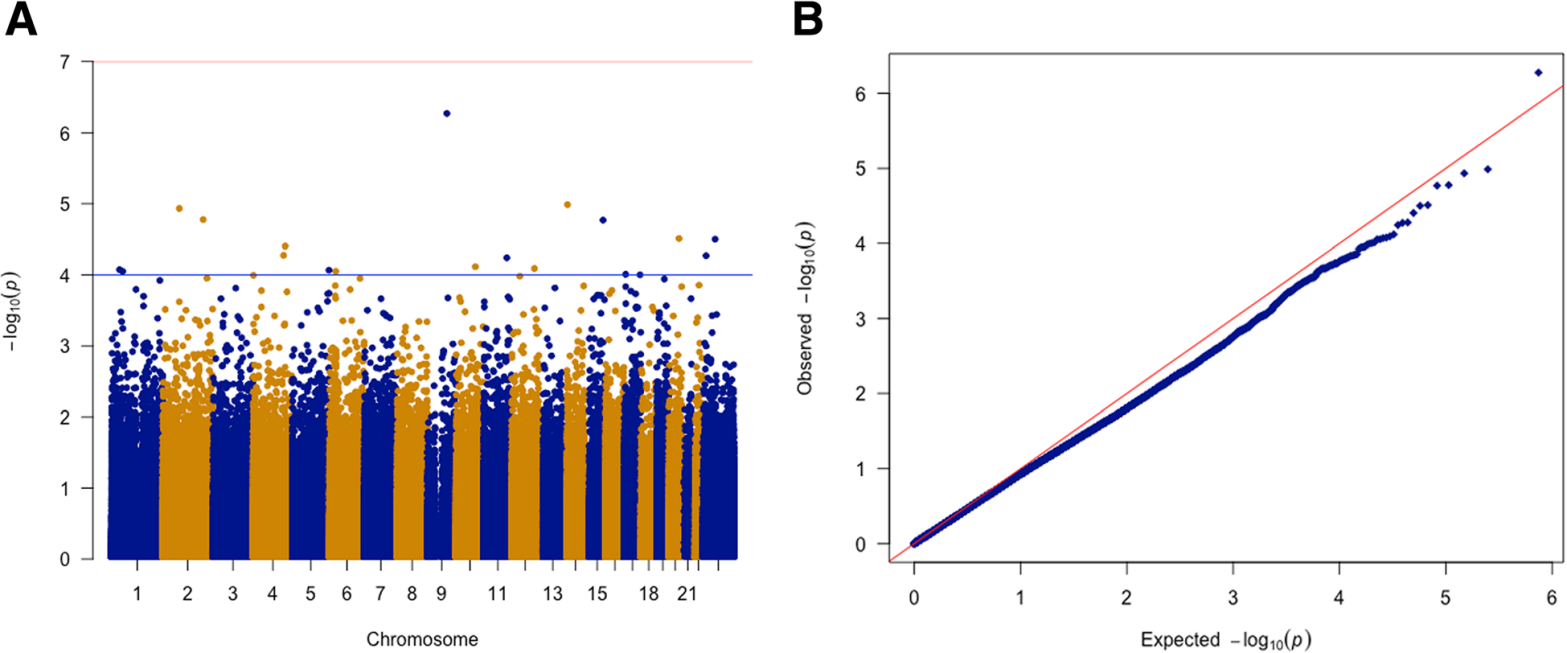
Manhattan plot and QQ plot for the SCDC MWAS. **a** Manhattan plot of the social and communication disorders (SCDC) MWAS. The blue line indicates the threshold of suggestive significance (*p* value < 1 × 10^−4^), and the red line indicates the threshold of statistical significance after multiple testing correction (1 × 10^−7^). **b** Quantile-Quantile plot of the SCDC MWAS

Methylome-wide association analysis (Methods, model 1) did not identify any significant loci after Bonferroni correction (*p* value < 1 × 10^−7^). The top CpG site was cg14379490, on chromosome 9 (MWAS beta = − 1.78 ± 0.35, *p* value = 5.34 × 10^*−*7^), which is equivalent to a 0.51 standard deviation unit decrease in SCDC scores. This CpG site is an “Open Sea” CpG site, whose closest gene is *FAM120A*, which encodes a scaffold protein that is expressed in a wide number of human tissues. We identified 19 CpG sites with suggestive *p* values (*p* value < 1 × 10^−4^) (Additional file 1: Table S2). The QQplot and the Manhattan plot are provided in Fig. 2. We did not find any evidence for inflation in *p* values (λ = 0.88), possibly because of the relatively small sample size and the regression model used. Further, gene-set analysis also did not identify significant association after correcting for multiple testing (Additional file 1: Table S3). To confirm that the results are robust to methodological differences, we re-ran the MWAS by using *M* values instead of beta values and using a different cell count estimate which included nucleated RBCs (Methods, model 2). There was a high correlation in Z-scores (*r* = 0.92, 95% CI = 0.92–0.92, *p* value < 2.2 × 10^−16^) between the two models. Subsequent analyses were conducted using model 1 MWAS results as these are easier to interpret.

To provide confidence to our primary analyses, we conducted an MWAS of scores on the CCC, which was reversed scored to identify difficulties in communication (Methods). The most significant CpG was cg13711424 (MWAS beta = − 3.73 ± 0.71, *p* value = 1.79 × 10^−7^), equivalent to a 0.55 standard deviation unit decrease in CCC scores. The Manhattan plot and QQ plot are included in Additional file 1: Figure S2. Of the 19 SCDC-associated CpGs of suggestive significance (*p* value < 1 × 10^−4^), the effect direction was concordant for 18 of them in the CCC MWAS (*p* value = 7.62 × 10^−5^, binomial sign test). Similarly, of the 32 CpGs with *p* value < 1 × 10^−4^ in the CCC MWAS, 28 had concordant effect direction in the SCDC MWAS (*p* value = 1.93 × 10^−5^, binomial sign test). Scores on the CCC and the SCDC were phenotypically correlated (*r* = 0.39, 95% CI = 0.32–0.45, *p* value < 2.2 × 10^−16^) in the participants who were included in the MWAS (*n* = 666), and both questionnaires measure difficulties in pragmatic communication. Given that we were testing correlated phenotypes in the same cohort using CpG methylation measured in the same tissue, we hypothesized that the MWAS for the two phenotypes will be positively correlated. The Z-scores for the MWAS of the two phenotypes were significantly correlated (*r* = 0.157, 95% CI = 0.153–0.160, *p* value < 2.2 × 10^−16^), which increased if we considered only CpGs with *p* value < 0.01 in either one of the phenotypes (P_SCDC_ < 0.01: *r* = 0.40, 95% CI = 0.36–0.43, *p* value < 2.2 × 10^−16^, P_CCC_ < 0.01: *r* = 0.40, 95% CI = 0.37–0.42, *p* value < 2.2 × 10^−16^).

### Enrichment analyses with peripheral blood methylation signatures

To investigate if there is an overlap between the SCDC MWAS and MWAS of autism conducted in peripheral tissues, we conducted effect direction concordance analysis with three autism MWAS datasets (MINERvA, SEED, and SSC, Methods). For all of them, we first investigated concordance in effect direction of all CpG sites with *p* value < 1 × 10^−4^. In contrast to the findings with the CCC MWAS, we did not identify a significant concordance in effect direction between the SCDC MWAS and any of the other three autism MWAS datasets. Comparing the three MWAS datasets to each other, we did not identify a significant concordance in effect direction for the suggestive CpGs in each of the comparisons (Table 1).

**Table 1:** Sign concordance of the SCDC MWAS and the three peripheral tissue MWAS at top loci (*p* value < 1 × 10^−4^)

		Testing dataset				Number of CpGs in the discovery dataset
		ALSPAC	MINERvA	SEED	SSC	
Discovery dataset	ALSPAC	*NA*	*10*	*11*	*4*	17
	MINERvA	*14*	*NA*	*17*	*19*	29
	SEED	*19*	*21*	*NA*	*18*	37
	SSC	*19*	*21*	*23*	*NA*	47

The table above provides the results of the tests for effect direction concordance. CpGs with *p* value < 1 × 10^−4^ in the discovery dataset were tested for concordance in effect direction in the testing dataset. The numbers in the cells (in italic) provides the total number of CpGs with concordant effect direction in the testing dataset. The number of CpGs in the discovery dataset provides the total number of CpGs in the discovery dataset with *p* value < 1 × 10^−4^. None of the results were significant (binomial sign test) after correcting for the multiple tests conducted

Given that there was limited evidence for concordance in effect direction between the datasets, we next tested if nominally significant CpGs (*p* value < 0.01) in the three autism MWAS have a shift toward lower *p* values in the SCDC MWAS using a one-sided Wilcoxon-rank sum test. This tests more CpGs than an effect direction concordance test and is agnostic to effect direction which may be discordant in different peripheral tissues measured at different developmental stages. After Bonferroni correction (alpha = 0.016), we did not identify a significant shift toward lower *p* values for the nominally significant CpGs from any of the three datasets (SEED: *p* value = 0.02; SSC: *p* value = 0.48; MINERvA: *p* value = 0.91), though we note a nominally significant shift in the SEED dataset. This lack of overlap may be due to the low statistical power of the MWAS of SSC and the three autism MWAS, none of which have identified significantly differentially methylated CpGs.

### Enrichment analyses with autism post-mortem brain methylation signatures

Methylation signatures in post-mortem brain tissues are more relevant to neurodevelopmental phenotypes than methylation signatures in peripheral tissue, and, for autism, are statistically better powered than MWAS in peripheral tissues, as these have identified more differentially methylated loci compared to peripheral tissue analyses [17, 20]. Considering this, we investigated if there is an overlap between the SCDC MWAS and MWAS of the post-mortem autism brain. Using data from the latest post-mortem brain study [20], we investigated concordance in effect direction between all CpG probes with *p* value < 1 × 10^−4^ from the cross-cortex analysis in the SCDC MWAS. Further, 171 out of 293 CpGs had a concordant effect direction in the two datasets (*p* value = 0.004). At a more stringent *p* value threshold of *p* value < 1 × 10^−5^, 88 of the 133 probes had concordant effect directions in the two datasets (*p* value = 2.4 × 10^−4^, binomial sign test). In contrast, Wilcoxon rank-sum test of all CpGs with *p* value < 0.01 in the post-mortem MWAS did not identify a significant shift toward lower *p* values (*p* value = 0.99, one-tailed Wilcoxon rank-sum test). We next tested if the results were supported in a different dataset. A previous study investigated differential methylation in post-mortem neurons from the frontal lobe (identified using FACS sorting) in autism [17]. First, testing effect direction concordance, 44 of the 87 CpGs with *p* value < 1 × 10^−4^ had concordant effect direction in the two datasets (*p* value = 1, binomial sign test). However, we identified a significant shift toward lower *p* values (*p* value = 9.3 × 10^−3^, one-tailed Wilcoxon rank-sum test) of all CpGs with *p* value < 0.01 in the SCDC MWAS.

### Enrichment with autism dysregulated genes

A few studies have identified consistent sets of dysregulated genes in autism, and co-expression modules enriched for these dysregulated genes [28–31]. Previous studies have identified a significant enrichment for differentially methylated autism CpGs in genes that are transcriptionally dysregulated in the post-mortem cortex in autism [14]. We investigated if CpGs mapped to transcriptionally dysregulated genes in the autism post-mortem cortex [40] and associated co-expression modules had a shift toward lower *p* values in the SCDC MWAS when compared to the other genes. We identified a significant shift toward lower *p* values for the transcriptionally dysregulated genes (one-sided Wilcoxon rank-sum test, *p* value = 6.22 × 10^−5^), but did not identify a significant enrichment for any of the modules (M4: *p* value = 0.58, M9: *p* value = 0.59, M16: *p* value = 0.042, M10: *p* value = 0.31, M20: *p* value = 0.42, M19: *p* value = 0.105).

### Genetic influences in SCDC methylation patterns

**Fig. 3:**
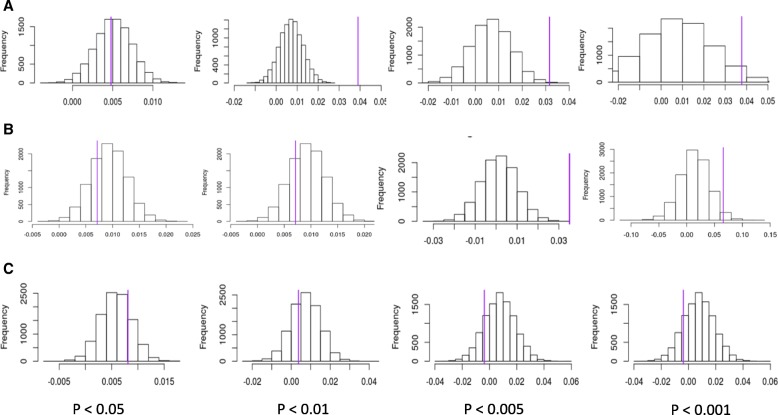
Permutation histogram of SNP-enrichment in top CpGs for three GWAS. The graphs present the results of the permutation analysis of the SNP enrichment. **a** Results of the autism GWAS. **b** Results of the SCDC GWAS. **c** Results of the Alzheimer’s GWAS. *p* value thresholds of the CpGs for enrichment are provided at the bottom of each column. *Y*-axis of each plot represents the frequency of the difference in mean p value of the mQTLs of CpGs below the threshold from the mean *p* value of the mQTLs of the CpGs above the threshold. *X*-axis represents the differences in the mean *p* value of the mQTLs of CpGs below the threshold from the mean *p* value of the mQTLs of the CpGs above the threshold. A higher difference in the means indicates a greater enrichment. Purple lines indicate the difference in mean of the non-permuted data point, i.e., the actual difference in mean

We next investigated if CpGs associated with SCDC scores are enriched for GWAS signals for autism. DNA methylation is under *cis* and, to a smaller extent, *trans* genetic control. We identified mQTLS associated with SCDC CpGs below four *p* value thresholds (P_SCDC_, Methods), and compared the distribution of *p* value of these mQTLS in the autism GWAS against the *p* value distributions of mQTLs above the P_SCDC_ (Methods). After multiple testing correction, mQTLS of CpGs with P_SCDC_ = 0.01, and 0.005 have significantly lower *p* values in the autism GWAS (P_SCDC_ 0.01: FDR-corrected *p* value = 5 × 10^−4^, P_SCDC_ 0.005, FDR-corrected *p* value = 4.75 × 10^−3^) (Table 2, Fig. 2). We provide additional support for this enrichment in a GWAS of SCDC, which is genetically correlated with autism. We identified an enrichment at P_SCDC_ 0.005 (FDR-corrected *p* value = 0.046) and at P_SCDC_ 0.001 (FDR-corrected *p* value = 0.046). In contrast, we did not identify an enrichment for mQTLs in the Alzheimer’s GWAS (Table 2, Fig. 3).

**Table 2:** Results of the enrichment analysis of the top CpGs

CpG p value threshold	GWAS	*p* value	Mean difference	FDR-corrected *p* value
0.05:	Autism	0.54	0.004	0.54
0.01:	Autism	1.0 × 10^−4^	0.039	5 × 10^−4^
0.005	Autism	1.9 × 10^−3^	0.031	4.75 × 10^−3^
0.001:	Autism	0.040	0.037	0.063
0.05:	SCDC	0.735	0.007	0.73
0.01:	SCDC	0.301	0.015	0.40
0.005	SCDC	0.022	0.038	0.046
0.001:	SCDC	0.023	0.065	0.046
0.05:	Alzheimer’s	0.343	0.008	0.853
0.01:	Alzheimer’s	0.710	0.003	0.853
0.005	Alzheimer’s	0.853	− 0.003	0.853
0.001:	Alzheimer’s	0.793	− 0.009	0.853

The table provides the results of the enrichment analyses for the top loci. We calculated the difference between the average *p* values for all the mQTLs mapped to CpGs below a selected threshold in the MWAS (CpG *p* value threshold) and the mQTLs mapped to CPGs above the threshold in the SCDC MWAS. This value is referred to as “mean difference” in the table. A positive difference suggests and enrichment. We then permuted the results after correcting for various factors, and computed a permuted *p* value (*p* value). We then corrected it for multiple testing using FDR correction (FDR-corrected *p* value). This was done using a GWAS for autism, SCDC, and Alzheimer’s

## Discussion

This study investigated the shared biology of autism and autistic traits by integrating genetic, methylation, and post-mortem gene expression data. We first investigated the validity of considering autistic traits for methylation studies. Considering autistic traits over a case-control design is useful in that (1) it captures greater variance across the underlying liability spectrum, (2) it can be used to increase sample sizes by phenotyping individuals for whom methylation data is available, and (3) it can be used to link methylation signatures from tissues collected in early life to the phenotype, as this can be more difficult for autism.

We conducted a prospective MWAS of autistic traits (SCDC) by measuring methylation signatures in the cord blood and linking it to autistic traits measured later in life. While we did not identify a significant CpG association with autistic traits after multiple testing correction, we were able to confirm that this analysis produced biologically meaningful signals by identifying significant correlation with an MWAS of a similar childhood phenotype (CCC) measured in the same cohort. Notably, the correlation in methylation values mirrored the phenotypic correlation. This, in principle, is similar to the idea of genetic correlation analysis of related phenotypes measured in the same cohort, though the methods used are very different.

Despite this, we did not identify a significant overlap between the MWAS of SCDC and MWAS of autism conducted using peripheral tissues [14, 16]. We note several differences between the SCDC MWAS analysis and the three MWAS for autism. Of primary importance is the statistical model used in the analysis. While we were interested in investigating if methylation signatures from cord blood were associated with SCDC scores measured in later life, all three peripheral tissue MWAS investigated if autism diagnosis was associated with differential methylation. Thus, in our analysis, methylation was an independent variable, whereas in the three MWAS for autism, methylation was a dependent variable. Second, there are remarkable differences in age at which methylation was measured, and confounding variables included in the analyses; for instance we included genetic principle components as covariates. Third, there are differences in tissue source as well. While the MINERvA cohort primarily used blood spots, the SEED and the SSC cohorts used whole blood for the respective MWAS. In comparison, our MWAS was conducted using cord blood. Fourth, while the SCDC MWAS was conducted in individuals of European ancestry, the SEED and SSC cohorts also included individuals of non-European ancestries. Fifth, while both the SCDC MWAS and the MINERvA MWAS had largely balanced sex ratio, the SEED and SSC cohorts had more male autistic individuals than females, though sex was included as covariate in these cohorts. None of the three autism MWAS demonstrated a significant overlap with each other as investigated using a sign-concordance test of the most significant CpGs. It is critical to investigate this observed lack of concordance, though it may be driven by the low statistical power of each individual MWAS, similar to the early GWAS studies which were underpowered.

In contrast to the results from the peripheral tissues, we observed some degree of overlap between MWAS conducted in post-mortem brain tissues [17, 20] and the SCDC MWAS. First, we found a significant sign concordance in CpGs identified in the largest cross-cortex MWAS of autism using post-mortem tissue samples. However, we did not identify an enrichment using a Wilcoxon rank-sum test of *p* values. In contrast, using a neuron-specific MWAS generated using a different post-mortem tissue dataset, we identified a significant overlap using a Wilcoxon-rank sum test of *p* values but not a significant sign-concordance. Additionally, using an RNA sequencing dataset of autism and neurotypical post-mortem brains [40], we identified a significant enrichment for transcriptionally dysregulated genes using a Wilcoxon rank-sum test. Overall, we are unable to strongly suggest that there is a significant overlap between the SCDC MWAS and the MWAS of autism in either post-mortem or peripheral blood tissues. This is likely due to multiple factors as outlined earlier. In addition, measuring methylation in peripheral tissue, which is not necessarily a relevant tissue for a neurodevelopmental condition like autism, is likely to attenuate the signal-to-noise ratio. Indeed, the post-mortem brain MWAS study [20] has identified significant CpGs with fewer samples compared with any of the three peripheral tissue MWAS [14, 16]. Thus, due to both the increased statistical power and the use of a relevant tissue, the top CpGs in the post-mortem brain MWAS are more likely to be true positives than the top CpGs in the peripheral tissue MWAS.

Given the highly polygenic nature of autism [11], it is likely that GWAS loci that are not statistically significant in the current GWAS studies may still influence methylation. Thus, the second aim of this study was to investigate if the top CpG sites in the SCDC MWAS were enriched for GWAS signals for autism and autistic traits. Our results demonstrate an enrichment for mQTLs for CpGs associated with SCDC scores in the GWAS for autism. We were able to provide additional support for the results in a much smaller GWAS of SCDC scores, but failed to identify an enrichment in a GWAS of Alzheimer’s [42], which is of comparable statistical power to the GWAS of autism. This enrichment is observed at more stringent *p* value thresholds providing confidence in our results. We did not test this in other peripheral tissue MWAS for which we had access to summary statistics given the lack of overlap between these and the SCDC MWAS.

### Limitations

Our study does not investigate causality. While methods such as Mendelian randomization can investigate causality [14, 49], this is typically restricted to a few number of loci based on current results of GWAS studies. In addition, we are restricted from using Mendelian randomization due to the low statistical power of both the MWAS and the GWAS datasets, resulting in the identification of a limited number of statistically significant loci. Two mechanisms may explain the overlap observed in the current dataset. The first is causal wherein genetic loci are likely to influence autism or autistic traits by influencing methylation of CpG sites, altering gene expression levels. The second is horizontal pleiotropy, where genetic loci are associated with autism or autistic traits, and separately, also influence methylation levels of CpG sites. This study cannot tease apart these two mechanisms.

A few caveats must be borne in mind while interpreting the results of this analysis. First, the current array-based method interrogates only a small proportion of all CpG sites in the genome. Thus, significant loci associated with autistic traits may lie outside of the regions interrogated. Second, due to the nature of the assay, the methylation values may also capture hydroxymethylation. We cannot exclude the possibility of signal attenuation due to assaying both hydroxymethylation and methylation in the current study, and the correlation between hydroxymethylation between blood and brain is low [50]. Third, while there is a modest but significant genetic and phenotypic correlation between autism and scores on the SCDC the SCDC only measures social aspects of autism and is not correlated with the non-social aspects of autism. Finally, age of gestation was not available to include as a covariate, and thus the current study does not account for it.

## Conclusions

Our study demonstrates a degree of methylomic overlap between autism and autistic traits, but we are limited in making further conclusions. Two factors—sample size and heterogeneity between the various samples—limit our understanding of methylation in autism. Future meta-analyses of both autism and autistic traits may help improve the statistical power of both the MWAS and aid in better understanding the shared etiology between the two phenotypes. We identified an enrichment for autism and autistic traits GWAS signals in the top CpG loci for autistic traits, but these must be replicated in independent MWAS of autistic traits.

## Additional file

Additional file 1: Supplementary Note. (DOCX 485 kb)
